# Exercise-Induced Fatigue in One Leg Does Not Impair the Neuromuscular Performance in the Contralateral Leg but Improves the Excitability of the Ipsilateral Corticospinal Pathway

**DOI:** 10.3390/brainsci9100250

**Published:** 2019-09-25

**Authors:** Saied Jalal Aboodarda, Cindy Xin Yu Zhang, Ruva Sharara, Madeleine Cline, Guillaume Y Millet

**Affiliations:** 1 Faculty of Kinesiology, University of Calgary, Calgary, AB T2N 1N4, Canada; saiedjalal.aboodarda@ucalgary.ca (S.J.A.); xinyu.zhang2@ucalgary.ca (C.X.Y.Z.); ruvarashe.sharara@ucalgary.ca (R.S.); madeleine.cline@ucalgary.ca (M.C.); 2 Inter-University Laboratory of Human Movement Biology, University of Lyon, UJM-Saint-Etienne, EA 7424, F-42023 Saint-Etienne, France

**Keywords:** cross-over fatigue, isometric contraction, force, voluntary activation, transcranial magnetic stimulation

## Abstract

To investigate the influence of pre-induced fatigue in one leg on neuromuscular performance and corticospinal responses of the contralateral homologous muscles, three experiments were conducted with different exercise protocols; A *(n* = 12): a 60 s rest vs. time-matched sustained left leg knee extension maximum voluntary contraction (MVC), B (*n* = 12): a 60 s rest vs. time-matched left leg MVC immediately followed by 60 s right leg MVC, and C (*n* = 9): a similar protocol to experiment B, but with blood flow occluded in the left leg while the right leg was performing the 60 s MVC. The neuromuscular assessment included 5 s knee extensions at 100%, 75%, and 50% of MVC. At each force level, transcranial magnetic and peripheral nerve stimuli were elicited to investigate the influence of different protocols on the right (tested) knee extensors’ maximal force output, voluntary activation, corticospinal excitability, and inhibition. The pre-induced fatigue in the left leg did not alter the performance nor the neuromuscular responses recorded from the right leg in the three experiments (all *p* > 0.3). However, enhanced corticospinal pathway excitability was evident in the tested knee extensors (*p* = 0.002). These results suggest that the pre-induced fatigue and muscle ischemia in one leg did not compromise the central and peripheral components of the neuromuscular function in the tested contralateral leg.

## 1. Introduction

Non-local muscle fatigue (NLMF) refers to a condition in which exercise-induced fatigue in a muscle group transiently impairs neuromuscular function in another limb [1]. In the absence of change in the contractile machinery of the non-exercised muscles (i.e., the index of peripheral fatigue), any attenuation in the neuromuscular function of these muscles is attributed to central mechanisms [2,3,4]. Presently, these central modulations are not well understood. However, it has been postulated that the fatiguing exercise-induced activation of group III and IV metabo- and mechano-receptors could compromise the motor control and execution of the non-fatigued muscles [5,6]. 

Transcranial magnetic stimulation (TMS) of the motor cortex in conjunction with electrical stimulation of the peripheral nerve (PNS) are non-invasive techniques that could be employed to investigate the central and peripheral mechanisms of fatigue [7]. Although several investigations have explored the influence of lower limb exercise-induced fatigue on responsiveness of the corticospinal pathway innervating upper limb muscles (heterologous muscle groups) [3,5,8], currently, there is little empirical evidence pertaining to the influence of a fatiguing exercise performed by a lower limb muscle group on central voluntary activation and corticospinal responses of the contralateral homologous muscles. 

A conventional research paradigm used to study the concept of NLMF includes a fatiguing exercise in one limb followed by the neuromuscular function assessment of the contralateral (non-fatigued) limb quantified during a single (4–6 s) maximum voluntary contraction (MVC) [9,10,11,12,13]. Given that in this approach the post-fatigue MVC is very brief, it has been postulated that participants might be able to overcome the sense of the pre-induced fatigue and maintain the MVC force output and central voluntary activation (VA) in the tested muscle group [4,10]. In an alternative paradigm, some investigators have recently used long-term sustained or intermittent MVCs performed by the tested muscle groups following the already fatigued contralateral limb [6,14,15]. The pre-induced fatigue in this approach has been shown to promote an augmented sense of fatigue and consequently, to attenuate the cycling performance in the tested contralateral limb [6]. However, there is little documented research that has studied this approach using sustained maximal isometric contractions. Halperin et al. [15] demonstrated a significant decline in the force output and VA (using the twitch interpolated technique) recorded during 12 (5 s) knee extension (KE) MVCs following a fatiguing exercise in the contralateral leg. On the contrary, Kennedy et al. [9] did not find any impairment in the MVC force, and VA recorded during 8 (2–3 s) KE MVCs. The controversy regarding these paradoxical findings was further fuelled by the fact that Kennedy et al. [9] maintained muscle ischemia in the fatigued leg to further activate the group III and IV muscle afferents, yet they did not find any change in MVC force and VA of the contralateral tested muscles. These findings argue against the idea that activation of group III and IV muscle afferents in one lower limb muscle could compromise the isometric performance in the contralateral limb. In addition to these controversies, none of the above-mentioned studies have explored the voluntary cortical activation (using TMS) nor the excitability and inhibition of the corticospinal pathway innervating the tested muscle groups following fatigue induced in the contralateral leg.

Thus, the purpose of the present study was threefold. In experiment A, we investigated whether fatiguing exercise in one leg (e.g., left) could alter the neuromuscular performance, corticospinal excitability, and inhibition of the tested (right) knee extensors recorded during brief maximal contraction performed immediately after the pre-induced fatigue. In experiment B, we explored whether pre-induced fatigue (in the left leg) combined with the subsequent sustained contraction (in the right leg) could alter the muscle performance and corticospinal responses in the tested (right) leg. In these two experiments, the group III and IV muscle afferents originating from the left knee extensors would stop firing after the termination of the sustained MVC in the leg [16]. Therefore, we hypothesized that the pre-induced left leg fatigue would not alter the neuromuscular performance recorded in the tested contralateral leg. In order to extend the input from group III and IV muscle afferents, we chose to maintain muscle ischemia in the fatigued (left) leg [16,17]. Thus, in experiment C, we investigated whether the prolonged activation of afferent receptors could modulate the neuromuscular performance and corticospinal responses in the tested (right) knee extensors. We hypothesized that maintaining blood ischemia in the fatigued leg could modulate the central motor drive and, consequently, the neuromuscular responses in the tested muscles.

## 2. Materials and Methods

### 2.1. Participants

Twelve healthy, recreationally active participants (six females: 22 ± 3 years, 168 ± 7 cm, 65 ± 1 kg; and six males: 25 ± 1 years, 175 ± 4 cm, 74 ± 9 kg) provided informed consent to participate in experiments A and B. Five participants from these two experiments and four additional participants volunteered to complete experiment C (three females: 26 ± 3 years, 169 ± 9 cm, 66 ± 9 kg; and six males: 30 ± 4 years, 173 ± 6 cm, 76 ± 8 kg). Participants did not have any history of neurological, cardiovascular, or musculoskeletal injuries and were determined as right-leg dominant based on the preferred leg used to kick a ball [18]. They were instructed to refrain from rigorous physical activity, as well as ingesting caffeine and alcohol at least 24 hours prior to each testing session. Participants also completed the TMS safety checklist [19] and Physical Activity Readiness Questionnaire for Everyone PAR-Q+ form [20]. The procedures were conducted in accordance with the declaration of Helsinki and approved by the Health Research Ethics Authority of the University of Calgary (REB17-0147). 

### 2.2. Experimental Setup and Procedures

The experimental protocol was performed on an isometric chair (Kin–Com, Chattecx Corporation, TN, USA) while the hips and knees were fixed at 90°. To ensure that the upper body did not contribute to the KE force, straps along the trunk and waist were secured, and participants crossed arms to shoulders during contractions. Left and right legs were each secured inside a padded ankle cuff attached to a strain gauge (LC101-2K; Omegadyne, Sunbury, OH, USA) with a non-extensible strap. Force data was sampled at 2000 Hz, digitally converted using the Power Lab acquisition hardware and Lab Chart software (ADInstruments, Bella Vista, Australia), and monitored on a computer. The electromyography (EMG) was recorded from the left and right knee-extensors, and both TMS and PNS were employed during the neuromuscular evaluations [21]. 

Bipolar self-adhesive Ag/AgCl surface EMG electrodes (Kendall MediTrace, MA) were positioned on the muscle belly of the right and left vastus lateralis (VL), rectus femoris (RF), and biceps femoris (BF) [22]. A reference electrode was placed on the patella of the right leg. Before the placement of electrodes, the area of skin was shaved and abraded to remove dead skin with sandpaper and cleaned with an isopropyl alcohol swab to decrease skin resistance. An inter-electrode impedance of <5 kΩ was obtained prior to recording to ensure an adequate signal-to-noise ratio. All EMG signals were digitized at a sampling rate of 2000 Hz by the PowerLab system (16/30-ML880/P, ADInstruments) and amplified with an octal bio-amplifier (ML138, ADInstruments). EMG signals were bandpass filtered (5–500 Hz) and all data were analyzed offline using the Labchart 8 software (ADInstruments).

Single-pulse TMS was manually delivered to the left motor cortex via a 110 mm concave double-cone coil (maximum output of 1.4 T) connected to a magnetic stimulator (Magstim 200^2^ Company Ltd., Whitland, UK). The optimal location of the coil was determined on the scalp for every centimeter from 2 cm anterior and 1 cm to the left of the vertex (i.e., the midpoint from nasal–inion and tragi). Single pulses of TMS at 50% of maximum stimulator output (MSO) were delivered during brief (2–3 s) knee extensions at 20% of MVC and the location which evoked the highest twitch force and motor evoked potential (MEP) amplitudes for VL and RF (without an increase in BF MEP amplitude) was marked on a latex swim cap worn by participants. The optimal stimulator intensity was determined by delivering four stimuli at each intensity between 20 to 80% of the MSO (with 10% increments, in random order) and the intensity that elicited the highest twitch force, as well as VL and RF MEP amplitudes, was used for the rest of the session. The size of superimposed twitch (SIT), VL, or RF amplitude showed a plateau up to 80% of MSO for all participants, thus further increase in TMS intensities (90% or 100% MSO) was not necessary. The average stimulating intensity was 61 ± 8% (range: 40%–80%) of the maximal stimulator output for the six testing sessions.

Square-wave percutaneous electrical stimuli were delivered to the femoral nerve using a constant current stimulator (DS7A, Digitimer, Welwyn Garden City, UK). The cathode electrode (Kendall MediTrace, MA, USA) was secured on top of the inguinal triangle, and the anode (50 × 90 mm electrode, Durastick Plus, CA, USA) was placed in between greater trochanter and suprailiac projections. The stimuli intensity (pulse duration: 1000 μs, maximal voltage: 400 V) was increased in incremental steps until a maximal twitch force and compound muscle action potential (i.e., VL and RF Mmax) were observed. The supramaximal stimulation (i.e., 130% of stimulator output that achieved maximal twitch and Mmax responses) was held constant throughout the protocol. The average stimulating intensity was 126 ± 52 mA (range: 65–185 mA) for the six testing sessions.

**Figure 1:**
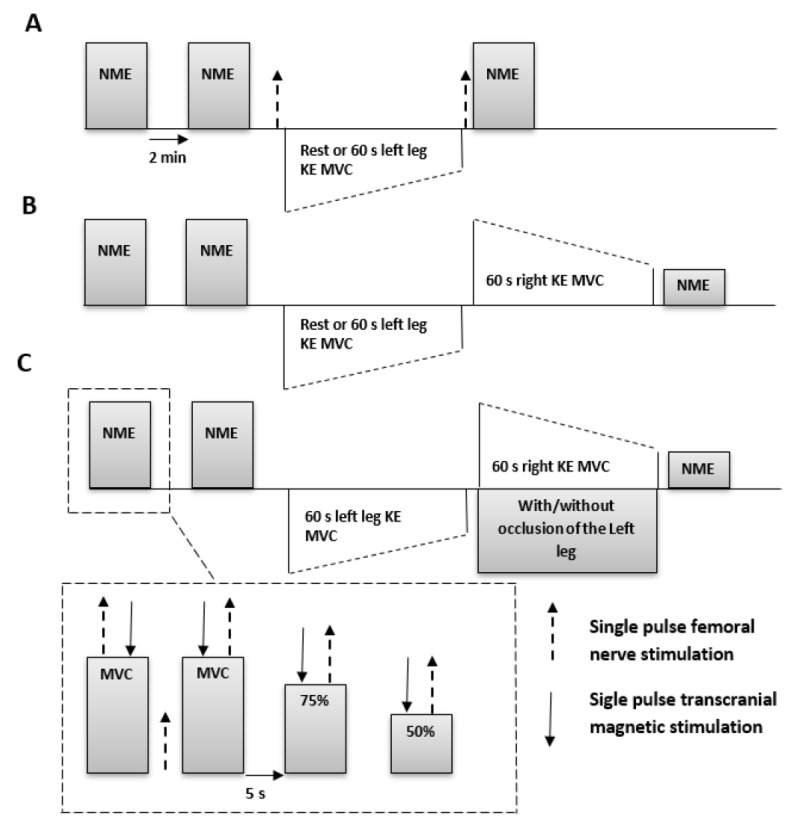
Details of experimental protocols. In experiment A (panel **A**) (*n* = 12), a 60 s rest or 60 s sustained left leg knee extension (KE) maximum voluntary contraction (MVC) were followed by a neuromuscular evaluation (NME) including 5 s sustained right leg KEs at 100%, 75%, and 50% of MVC. In experiment B (panel **B**) (*n* = 12), a 60 s rest or 60 s left leg MVC was immediately followed by a 60 s right leg MVC. In experiment C (panel **C**) (*n* = 9), a similar protocol as experiment B was performed while blood flow was occluded in the left leg while the right leg was performing a 60 s MVC.

Experiment A (*n* = 12). Neurophysiological responses recorded immediately after a contralateral fatiguing exercise. In two randomly selected testing sessions, the fatigued (left) leg underwent either 60 s sustained KE MVC (Fatigue; Ftg-0) or 60 s rest (Rest-0) (Figure 1A). Before and immediately after these two protocols, the tested (right) leg performed a neuromuscular evaluation including a 5 s KE MVC superimposed with a PNS delivered on the force plateau and another PNS evoked 2 s after the MVC on the relaxed muscle (i.e., potentiated twitch, Pt). Three seconds after the Pt, subjects performed another 5 s MVC followed by contractions at 75% and 50% of MVC. Single-pulse TMS and PNS were delivered at 100%, 75%, and 50% of MVC with ~2 s interstimulus intervals, during which participants would reobtain the target force following each TMS interference to the voluntary force output. The resting interval between 100%, 75%, and 50% MVC was ~5 s. Two sets of neuromuscular evaluations were performed before and one set was performed immediately after each intervention. Additionally, immediately before and after each left leg protocol (either rest or 60 s sustained MVC), a PNS was evoked to the right leg at rest to account for any potential influence of contralateral protocols on contractile property and excitability of the tested muscles.

Experiment B (*n* = 12). Neurophysiological responses recorded after a sustained MVC superimposed upon the pre-induced contralateral fatigue. In two randomly selected testing sessions (Figure 1B), participants undertook 60 s rest (Rest-60) or 60 s left leg sustained MVC (Ftg-60), followed immediately by 60 s sustained right leg MVC. Neuromuscular evaluations, as described in experiment A, were performed before and immediately after each experimental protocol.

Experiment C (*n* = 9). Influence of maintaining muscle ischemia in the fatigued contralateral leg on the neurophysiological responses of the tested muscles. In two randomly selected testing sessions, participants underwent the Ftg-60 protocol (see experiment B) with the exception of the “cuff session (Ftg-60-Occl), where blood flow to the left (fatigued) leg was occluded 2 s prior to the end of 60 s MVC (Figure 1C). A standard single-bladder adult thigh cuff connected to an automatic rapid inflation system (Hokanson E20 AG101, Bellevue, WA, USA) was located at the distal part of the thigh. The cuff was inflated to 300 mmHg using compressed air within 2 s. 

### 2.3. Data Analysis

*Performance.* A force-time integral including the sum of the data points, multiplied by the sample interval during the 60 s sustained MVCs (performed by the tested leg) was calculated in experiments B and C.

*Neuromuscular function.* The maximal force output and the corresponding root mean square EMG [rmsEMG of the VL and RF muscles] was quantified during the first and last 2 s intervals of the sustained 60 s left and right leg MVCs, as well as over 500 ms prior to each TMS evoked during brief right leg MVCs. The VL and RF rmsEMG were normalized to the amplitude of the corresponding muscle compound action potential (Mmax) recorded during the same contraction to calculate the rmsEMG∙Mmax^−1^ ratio (rmsEMG_100_). 

The central voluntary activation (VA_PNS_) was measured using the twitch interpolation technique [23] with the following formula, where Fb is the voluntary force output just before the PNS, D is the difference between Fb and the maximum SIT force evoked by the stimulus, F_MAX_ is the highest force output during MVC, and FPt is the size of Pt force evoked at rest [24].

VA (%) = 100 − D × (Fb/F_MAX_)/FPt × 100 (1)

The voluntary cortical activation (VA_TMS_) was assessed using the amplitude of SITs evoked by TMS during 5 s contractions at 100%, 75%, and 50% of MVC [25,26]. The *y*-intercept of the linear regression (*r*^2^ > 0.9) between the SITs was computed to identify the estimated resting twitch (ERT). VA_TMS_ was then calculated using the following equation:

VA_TMS_ = [1 − (SIT/ERT)] × 100 (2)

In order to explore the excitability of the corticospinal pathway, the areas under the MEP and the Mmax signals were measured for both VL and RF muscles. The onset of MEP and Mmax were defined as the point at which the voltage trace became tangential to baseline in either the positive or negative direction. The responses to VL and RF MEP were normalized to the subsequent Mmax recorded during the same contraction to calculate MEP∙Mmax^-1^ ratios (MEP_100_, MEP_75_, MEP_50_). The duration (ms) of the silent period (i.e., indicative of corticospinal inhibition) was assessed for VL and RF MEPs as the interval from the stimulus artifact to the return of the continuous EMG by visual inspection during 100%, 75%, and 50% of MVC contractions (SP_100_, SP_75_, SP_50_). The end of the SP for two participants in experiments A and B was not distinguishable, so the values were removed from the data pool. 

### 2.4. Statistical Analysis

**Figure 2:**
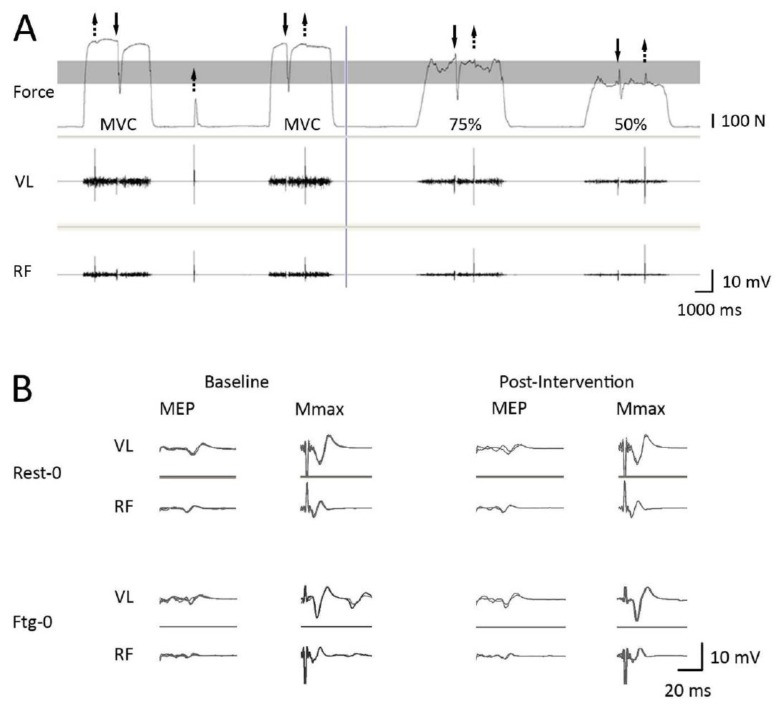
Representative traces from a single subject for the force output and VL and RF EMG signals at 100%, 75%, and 50% of MVC (panel **A**) and the MEPs and Mmaxs recorded from VL and RF at 100% of MVC at baseline and post-intervention levels following a 60 s rest (Rest-0) or 60 s knee extension MVC with the contralateral limb (Ftg-0) (panel **B**). The top and the bottom line of the shaded grey box (in panel **A**) represents 75% and 50% of MVC, respectively. In panel **B**, four MEPs and four Mmaxs recorded at baseline and two MEPs and two Mmaxs recorded at the post-intervention level are superimposed (see Figure 1 for more details).

**Figure 3:**
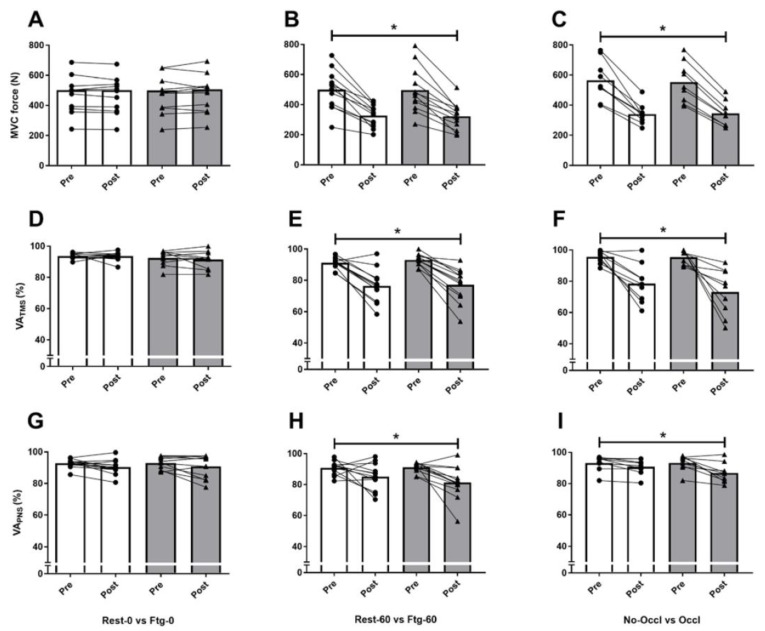
Mean and SD of knee extension (right leg) maximum voluntary contraction (MVC), voluntary activation (VA) using transcranial magnetic stimulation (VA_TMS_), and peripheral nerve stimulation (VA_PNS_) values normalized to the baseline. In experiments A (panels **A**,**D**,**G**), a 60 s rest (Rest-0) vs. a 60 s left leg MVC (Ftg-0) were compared. In experiment B (panels **B**,**E**,**H**), a 60 s rest (Rest-60) or a 60 s left leg MVC (Ftg-60) followed immediately by a 60 s right leg MVC were compared. In experiment C (panels **C**,**F**,**I**), Ftg-60 was performed at the absence or presence of a blood flow occlusion in the left leg (Ftg-60-Occl). * Significantly different from baseline (time effect).

**Figure 4:**
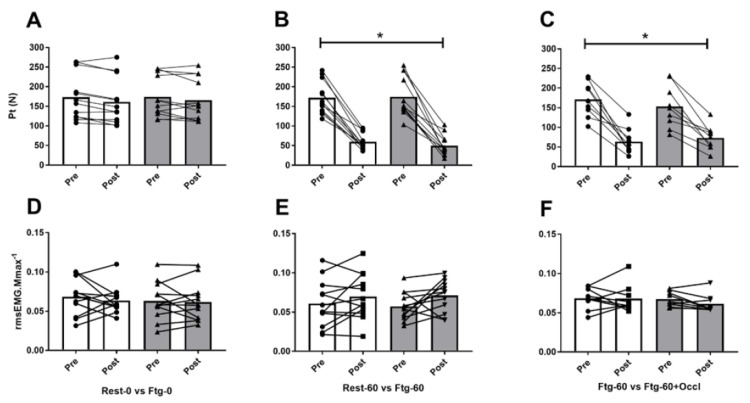
Mean and SD of potentiated twitch (Pt) and rectus femoris (RF) root mean square EMG (rmsEMG) values normalized to the baseline. In experiments A (panels **A**,**D**), a 60 s rest (Rest-0) or a 60 s left leg MVC (Ftg-0) were compared. In experiment B (panels **B**,**E**), a 60 s rest (Rest-60) or a 60 s left leg MVC (Ftg-60) followed immediately by a 60 s right leg MVC were compared. In experiment C (panels **C**,**F**), Ftg-60 was performed at the absence or presence of a blood flow occlusion in the left leg (Ftg-60-Occl). * Significantly different from baseline (time effect).

**Figure 5:**
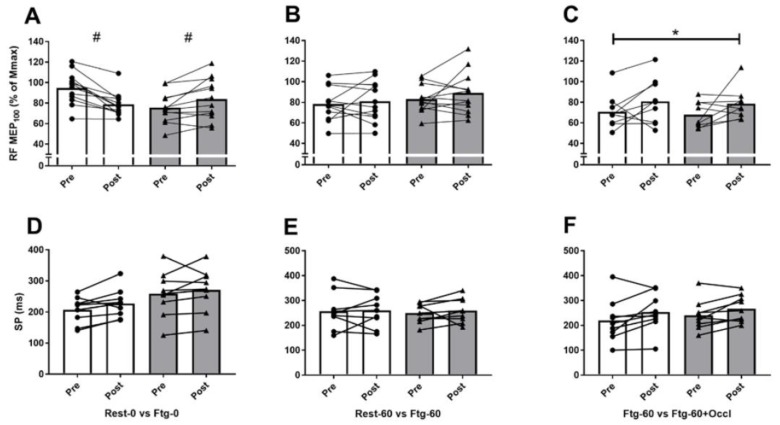
Mean and SD of motor evoked potential normalized to the subsequent muscle compound action potential (MEP∙Mmax^−1^ [MEP]) and the duration of silent period (SP) recorded from the rectus femoris (RF) during MVC. The values are presented as a percentage of baseline. In experiments A (panels **A**,**D**), a 60 s rest (Rest-0) or a 60 s left leg MVC (Ftg-0) were compared. In experiment B (panels **B**,**E**), a 60s rest (Rest-60) or a 60 s left leg MVC (Ftg-60) followed immediately by a 60 s right leg MVC were compared. In experiment C (panels **C**,**F**), Ftg-60 was performed in the absence or presence of a blood flow occlusion in the left leg (Ftg-60-Occl). * Significantly different from baseline (time effect), # significantly different from baseline (interaction effect).

Statistical analyses were computed using SPSS software (version 23.0; SPSS, Inc., Chicago, IL, USA). Shapiro–Wilk and Mauchly tests were used to ensure the assumptions of normality and sphericity for all dependent variables, respectively. All variables were normally distributed. Greenhouse–Geisser correction factor was applied when the assumption of sphericity was violated. Unless otherwise notified, sphericity assumed data were reported. For each experiment separately, a paired sample *t*-test was used to compare baseline measures and then a two-way repeated measure analysis of variances (ANOVA) was run to explore the effect of 2times (baseline vs. post-intervention values) × 2 conditions for all outcome variables. When ANOVAs showed significant main effects of times or conditions, Bonferroni post hoc test was used to compare values. When an interaction effect was observed, paired *t*-tests with Holm–Bonferroni corrections were applied. In one case where baseline measures revealed a significant difference between conditions (i.e., MEP_100_ in experiment A), the post-intervention values were normalized to the baseline (to account for the day to day variations), and then the paired *t*-test was used to compare the relative values between different conditions. Paired *t*-test was used to compare the force-time integral values (calculated during the 60 s MVCs performed by the tested leg) between the two conditions in each experiment. The effect size was calculated for repeated measure ANOVAs by converting partial eta-squared to Cohen’s d and for paired comparisons by using the mean ± SD values [27]. According to Cohen [27], the magnitude of effect size was classified as small (0.2 ≤ d < 0.5), medium (0.5 ≤ d < 0.8), and large (d ≥ 0.8). The mean ± SD of variables are presented in the text, Figure 1, Figure 2, Figure 3, Figure 4 and Figure 5, and the Table.

## 3. Results

*Force changes during left (fatigued) leg sustained MVCs.* The decline in the maximal force output recorded during the 60 s left KE MVC in three experiments was: −55% (mean ± SD: 471 ± 137 to 209 ± 67 N) following Ftg-0 in experiment A, −54% (477 ± 133 to 218 ± 54 N) following Ftg-60 in experiment B, and −51% (529 ± 139 to 260 ± 35 N), and −56% (571 ± 170 to 251 ± 93 N) after Ftg-60 and Ftg-60-Occl in experiment C, respectively. 

### 3.1. Experiment A

The pre-induced fatigue in the contralateral (left) leg did not alter MVC, rmsEMG_100_, Pt, Mmax, VA_PNS_, and VA_TMS_ recorded from the right (tested) leg in the Ftg-0 compared to Rest-0 condition (Figure 3A,D,G, and Figure 4A,D). The resting twitch and corresponding Mmax evoked before and after 60 s left leg MVC (Figure 1A and Figure 2B) also did not show any difference between the two conditions (data not shown). However, a significant interaction effect of time × group was observed for the corticospinal excitability (MEP∙Mmax^−1^ ratio) recorded from VL (F_1,11_ = 6.29, *p* = 0.031, d = 1.58), and RF (F_1,11_ = 13.33, *p* = 0.004, d = 0.84) at 100% MVC (MEP_100_). While, higher values were observed for VL (*p* = 0.032, d = 0.39) and RF (*p* = 0.020, d = 0.43) following the contralateral leg fatigue (in Ftg-0), a decline was observed for the resting condition (in Rest-0) (Figure 5A). 

### 3.2. Experiment B

As expected, the MVC force (F_1,11_ = 50.9, *p* < 0.001, d = 4.29), Pt (F_1,11_ = 109.3, *p* < 0.001, d = 6.32), VA_PNS_ (F_1,11_ = 7.21, *p* = 0.023, d = 1.69) and VA_TMS_ (F_1,11_ = 27.7, *p* = 0.001, d = 3.71) significantly declined (all time effects) following 60 s right leg MVC. However, the pre-induced fatigue in the left leg did not result in any significant difference in the neuromuscular performance and corticospinal excitability and inhibition measures between Rest-60 and Ftg-60 conditions (Figure 3B,E,H, Figure 4B,E and Figure 5B,E). 

### 3.3. Experiment C

The VA_TMS_ values from one participant were removed from the data pool because the linear regression line exhibited *r^2^* < 0.9. Similar to experiment B, the MVC force (F_1,8_ = 84.3, *p* < 0.001, d = 6.13), Pt (F_1,8_ = 47.4, *p* < 0.001, d = 4.87), VA_PNS_ (F_1,8_ = 16.8, *p* = 0.005, d = 3.09) and VA_TMS_ (F_1,7_ = 16.8, *p* = 0.003, d = 2.90) significantly declined and MEP_100_ (F_1,8_ = 29.9, *p* = 0.001, d =1.49) increased (all time effects) following 60 s right leg MVC. However, the pre-induced fatigue and the subsequent blood occlusion in the left leg (while the right leg was performing the 60 s MVC) did not result in any significant difference in the neuromuscular performance and corticospinal excitability and inhibition between Ftg-60 and Ftg-60-Occl conditions (Figure 3C,F,I, Figure 4C,F and Figure 5C,F). 

The left leg pre-induced fatigue in experiments B and C did not alter the force-time integral values recorded across 60 s sustained right leg MVCs between Rest-60 (19,737 ± 6847 N∙s) vs. Ftg-60 (18,264 ± 7413 Ns) as well as the Ftg-60 (22,422 ± 3645 Ns) vs. Ftg-60-Occl (22,744 ± 4197 Ns). In addition, the corticospinal excitability (MEP) and inhibition (SP) recorded from VL and RF during contractions at 100%, 75%, and 50% of MVC did not demonstrate any significance in the three experiments unless otherwise stated above. Since VL and RF rmsEMG, MEP, and SP demonstrated similar patterns, only the results of RF were presented in Figure 3, Figure 4 and Figure 5.

## 4. Discussion

The most important findings of the present study are that (i) in line with our first hypothesis, the pre-induced left leg fatigue did not alter the right knee extensors neuromuscular function, while (ii) the VL and RF corticospinal excitability (MEP_100_) significantly increased following the 60 s contralateral MVC (in Ftg-0) compared to a time-matched rest (in Rest-0), and (iii) contrary to our second hypothesis, the pre-induced fatigue, in combination with the subsequent left leg blood occlusion did not alter the neuromuscular function or performance (i.e., force-time integral during 60 s MVC) nor corticospinal responses between Ftg-60-Occl vs. Ftg-60 condition. These results confirm that 60 s sustained MVC-induced fatigue and subsequent muscle ischemia in one lower limb muscle, activating the group III and IV muscle afferents, does not compromise the neuromuscular performance in the contralateral limb when performed in isometric conditions.

### 4.1. MVC Force and Central Motor Drive

Multiple studies have found that exercise-induced fatigue in one leg may impair the neuromuscular function in the contralateral leg (for review see [1]). However, the results of the current study, in line with several other investigations [9,10,11,12,13], have failed to show the NLMF effect in the lower limb muscles. This lack of observable NLMF is apparent in a research paradigm where the post-intervention neuromuscular function is assessed during a single, brief (4–6 s) MVC [6,11,28,29]. It was hypothesized that in this paradigm, despite a general sensation of fatigue created by the pre-induced fatiguing task, participants would be able to maintain the level of central motor commands to the contralateral tested muscles to prevent a deterioration in maximal performance. In line with this hypothesis, the indices of central fatigue, including rmsEMG_100_ as well as the central neural drive (measured by VA_PNS_) and neural drive associated with circuits at or above the cortical motor cells (measured by VA_TMS_), did not change following Ftg-0 and Rest-0 conditions. These findings confirm that a 60 s MVC in one leg does not result in any central constraint to the performance of the tested contralateral muscles when the post-fatigue assessment includes a brief MVC [4,30].

In an alternative research paradigm, Halperin et al. [4,15] demonstrated that MVC force and VA_PNS_ were compromised when 12 × 5 s MVCs (as opposed to a single MVC) were performed following the fatiguing contralateral limb. Contrary to their observation, however, Kennedy et al. [9] did not find any impairment in the MVC force and VA_PNS_ recorded during 8 × 3 s MVCs, despite the fact that these investigators maintained muscle ischemia in the fatigued leg to further activate the group III and IV muscle afferents. The results of our study (in experiments B and C) support those reported by Kennedy et al. [9] indicating that adding 60 s sustained MVC in combination with subsequent muscle ischemia to the contralateral leg does not alter the central (rmsEMG_100_, VA_PNS_, and VA_TMS_) and peripheral (Pt and Mmax) indices of fatigue in the tested limb. Of note, in experiments B and C, while VA_TMS_ demonstrated 18 to 25% reduction (Table 1, Figure 3E,F), only 3% to 10% decrement was observed for VA_PNS_ (Table 1, Figure 3H,I). Although one may take these results to suggest that different sites within the central nervous system would play distinct roles in impaired maximal force output after the fatiguing task, limitations associated with the interpretation of the voluntary activation data [31] make it difficult to draw an explicit conclusion about the results. Nonetheless, pre-induced fatigue and muscle ischemia in the contralateral limb did not alter the contribution of cortical and motoneuronal circuits in the end-exercise central fatigue level. 

As opposed to previous experiments that used intermittent brief MVCs as the fatiguing task for the tested leg [9,15], we used 60 s sustained MVC to generate high intramuscular pressure and decreased oxygen delivery due to muscle ischemia induced by sustained MVC [32]. The 60 s MVC was chosen to match the total duration of intermittent MVCs (12 × 5 s MVCs) used by Halperin et al. [15]. However, the left leg pre-induced fatigue in combination with the blood ischemia did not alter the force-time integral values recorded during the 60 s right leg sustained MVCs. These results suggest that the activation of group III and IV muscle afferents induced by a 60 s sustained MVC in one leg does not modulate the motor performance in the contralateral leg during either sustained or brief MVCs. 

A point of deliberation in the present study may be that the magnitude of the pre-induced fatigue generated by the left leg was not enough to constitute an NLMF. Indeed, two 100 s sustained KE MVCs used by Halperin et al. [15] resulted in ~70% force drop in the fatigue limb whereas one 60 s MVC resulted in ~55% reduction in MVC in our study. However, the neuromuscular performance was not altered in the study by Kennedy et al. [9] despite a 77% decline in MVC force. We also minimized the time delay between the fatiguing tasks and the post-intervention neuromuscular assessments to avoid recovery of central and peripheral fatigue, yet no evidence of NLMF was found. This time delay in previous studies was approximately 15–60 s [9,15]. It is also worth clarifying that the failure in observing NLMF phenomenon in the current study might be associated with the size of muscle mass involved (i.e., knee extensors in one leg) and the mode of exercise (i.e., sustained MVC) performed during the fatiguing task. Prior investigations that used bilateral dynamic knee extensions [8], leg cycling [5], and arm cranking exercises [32] found NLMF in the rested/remote muscle groups. The accelerated neuromuscular fatigue within the tested muscle groups in these studies have been attributed to central inhibitory factors mediated by afferent feedback from pre-fatigued muscles. Nonetheless, further studies using a longer duration of sustained MVC and larger muscle mass during whole-body dynamic exercises are required to explore the influence of group III and IV muscle afferents on the NLMF phenomenon. 

### 4.2. Corticospinal Excitability and Inhibition

The corticospinal pathway excitability recorded from RF and VL muscles demonstrated relatively greater values (MEP_100_) following 60 s contralateral MVC (in Ftg-0) compared to a time-matched rest (in Rest-0). Although the current study showed a small effect size, this so-called “cross-over facilitation” of the corticospinal pathway had previously been shown in the upper limb musculature [33] and was attributed to (i) transfer of excitatory signals from one hemisphere to another via the callosal commissure [34,35], (ii) increase in the excitability of motoneurons innervating the tested muscles via release of monoaminergic neuromodulators [36], and (iii) activation of additional brain regions including ipsilateral and contralateral prefrontal and sensorimotor areas as a compensatory mechanism to prevent reduction in maximal force output [37]. With the single-pulse TMS used in the present study, it is indeed difficult to confirm the contribution of the above-mentioned mechanisms; particularly because the MEP_75_ and MEP_50_ recorded from both VL and RF did not show an increase following contralateral fatiguing contraction. However, in support of these notions contributing to cross-over facilitation of MEP recorded during MVC (MEP_100_), Tanaka and Watanabe [37] have suggested that additional brain regions are involved in a task if participants choose to mobilize all of their mental effort to execute a “maximal” rather than a “submaximal” contraction. 

The failure in observing cross-over facilitation of MEP_100_ following Ftg-60 (experiment B) and Ftg-60-Occl (experiment C) is not clear; however, this could be attributed to the 60 s right leg MVC. More specifically, the corticospinal excitability (i.e., MEP_100_) demonstrated a significant increase following the 60 s sustained right leg MVC (time effect) (Figure 5B,C). This MEP facilitation was observed regardless of the preceding conditions undertook by the left leg (i.e., 60 s MVC vs. 60 s MVC + occlusion in experiment C). Thus, our data suggest that the 60 s sustained MVC performed by the right (tested) knee extensors facilitated a strong excitatory effect to the corticospinal system while the contralateral fatiguing contraction did not enhance this facilitation any further. 

Our data in all three experiments demonstrated no statistical difference in the duration of SP compared within or between conditions. This finding indicates that the pre-induced fatigue and the subsequent blood ischemia in one leg do not alter the rate of corticospinal inhibition in the contralateral homologous muscles recorded during 100%, 75%, and 50% of MVC contractions.

### 4.3. Limitations

Methodological considerations of the present study include: (i) The sequence of contractions in the neuromuscular assessment (i.e., 100%, 75%, and 50% of MVC) was kept consistent throughout the experiment whereas an early recruitment of high threshold motoneurons during the MVC could have offset the influence of contralateral contraction on MEP_75_ and MEP_50_, (ii) the size of MEP monitors the excitability of the pyramidal tract neurons as well as the spinal motoneurons and spinal interneurons [38]; thus, further research is required to elucidate the influence of pre-induced fatiguing exercises on the cortical and spinal excitability responses, (iii) some may argue that the antagonist muscle excitability (M-wave amplitude) and contractile property (peak twitch) might have affected the slope of the regression line used to calculate the estimated resting twitch in VA_TMS_ measurement, however, the hamstring Mmax and peak twitch were not measured due to complexity of the sciatic nerve electrical stimulation; therefore, caution should be taken in interpretation of the VA_TMS_ data, as it is difficult to determine the contribution of hamstring coactivation on TMS-evoked knee extensors SITs, (iv) pre-induced fatiguing exercise in one limb may modulate central and peripheral homodynamic associates such as cardiorespiratory responses, neurotransmitters, neuromodulators, hormonal factors as well as autonomic responses, however, these parameters were not directly measured in the current study, thus they were not discussed, and finally, (v) more investigation should be directed towards the measurement of intracortical facilitation and inhibition using the paired-pulse TMS paradigm. 

## 5. Conclusions

In conclusion, the results of the present study suggest that the pre-induced fatigue evoked by the 60 s sustained KE MVC, with or without subsequent blood ischemia, in the fatigued leg does not compromise the performance and neuromuscular function of the tested contralateral leg. However, the pre-induced fatigue may enhance the responsiveness of the ipsilateral corticospinal pathway innervating the tested limb, provided that the neuromuscular evaluation in the tested muscles is performed immediately after the pre-induced fatigue.

## Figures and Tables

**Table 1:** Group data (mean and SD) for variables recorded from the knee extensor muscles during 100%, 75%, and 50% of MVC in the three experiments.

Variables		Experiment A		Experiment B		Experiment C	
		Rest-0	Ftg-0	Rest-60	Ftg-60	Ftg-60	Ftg-60-Occl
MVC force (N)	Baseline	501.7 ± 159.9	499.9 ± 158.7	507.1 ± 148.2	495.3 ± 150.3	555.0 ± 128.8	573.9 ± 144.1
	Post-test	501.1 ± 170.7	506.3 ± 158.6	334.8 ± 88.9	322.2 ± 89.9	349.9 ± 71.1	363.6 ± 96.7
rmsEMG∙Mmax^−1^ ratio (rmsEMG_100_)	Baseline	0.074 ± 0.031	0.070 ± 0.037	0.067 ± 0.034	0.066 ± 0.026	0.063 ± 0.030	0.068 ± 0.026
	Post-test	0.069 ± 0.028	0.069 ± 0.044	0.066 ± 0.026	0.078 ± 0.029	0.066 ± 0.031	0.072 ± 0.038
PT (N)	Baseline	173.2 ± 54.4	173.7 ± 51.7	171.8 ± 41.7	174.5 ± 50.4	171.5 ± 44.3	153.4 ± 54.5
	Post-test	161.4 ± 59.1	165.6 ± 52.9	59.9 ± 20.2	49.8 ± 26.1	63.5 ± 33.2	72.8 ± 31.1
VA_PNS_ (%)	Baseline	92.7 ± 2.9	93.1 ± 3.8	90.7 ± 4.5	91.1 ± 3.2	93.1 ± 5.1	93.2 ± 5.2
	Post-test	90.6 ± 4.9	90.7 ± 7.5	85.1 ± 9.1	81.2 ± 11.2	90.6 ± 5.2	86.7 ± 6.7
VA_TMS_ (%)	Baseline	93.6 ± 1.9	92.6 ± 5.2	91.1 ± 4.2	93.1 ± 4.4	94.8 ± 5.5	85.4 ± 4.5
	Post-test	93.4 ± 3.1	91.2 ± 6.1	73.3 ± 18.3	76.6 ± 12.6	78.4 ± 12.2	70.1 ± 17.1
RF MEP∙Mmax^−1^ (MEP_100_)	Baseline	0.93 ± 0.18	0.80 ± 0.21	0.77 ± 0.18	0.83 ± 0.14	0.70 ± 0.16	0.67 ± 0.12
	Post-test	0.82 ± 0.18 #	0.90 ± 0.25 #	0.79 ± 0.21	0.88 ± 0.21	0.80 ± 0.22	0.78 ± 0.22
RF MEP∙Mmax^−1^ (MEP_75_)	Baseline	0.94 ± 0.19	0.84 ± 0.23	0.87 ± 0.20	0.92 ± 0.15	0.82 ± 0.15	0.81 ± 0.15
	Post-test	0.87 ± 0.22	0.82 ± 0.20	0.83 ± 0.22	0.89 ± 0.11	0.92 ± 0.24	0.84 ± 0.33
RF MEP∙Mmax^−1^ (MEP_50_)	Baseline	0.96 ± 0.15	0.90 ± 0.19	0.89 ± 0.18	0.97 ± 0.13	0.89 ± 0.14	0.81 ± 09
	Post-test	0.95 ± 0.16	0.88 ± 0.19	0.87 ± 0.14	0.88 ± 0.22	0.97 ± 0.34	0.79 ± 0.28
RF SP_100_ (ms)	Baseline	206.7 ± 45.2	258.5 ± 78.3	257.5 ± 78.6	249.3 ± 41.6	219.1 ± 84.6	240.6 ± 60.5
	Post-test	227.3 ± 50.0	270.9 ± 74.7	260.1 ± 69.7	259.3 ± 52.0	253.4 ± 75.3	267.4 ± 53.7
RF SP_75_ (ms)	Baseline	211.4 ± 48.3	256.8 ± 59.6	267.7 ± 64.0	252.0 ± 54.3	214.1 ± 77.7	208.5 ± 96.8
	Post-test	226.1 ± 67.3	270.3 ± 77.0	254.1 ± 70.3	254.9 ± 56.5	231.6 ± 67.5	250.2 ± 55.9
RF SP_50_ (ms)	Baseline	231.6 ± 40.9	276.4 ± 61.4	270.0 ± 69.1	273.1 ± 45.9	207.3 ± 71.8	213.1 ± 47.4
	Post-test	248.9 ± 63.2	269.8 ± 61.2	256.9 ± 61.2	261.5 ± 55.6	248.5 ± 74.6	253.2 ± 77.3
BF MEP_100_ (mV.s)	Baseline	0.009 ± 0.005	0.010 ± 0.008	0.007 ± 0.004	0.009 ± 0.005	0.008 ± 0.005	0.008 ± 0.004
	Post-test	0.007 ± 0.006	0.009 ± 0.006	0.007 ± 0.005	0.007 ± 0.005	0.008 ± 0.005	0.006 ± 0.005
BF MEP_75_ (mV.s)	Baseline	0.007 ± 0.005	0.009 ± 0.008	0.007 ± 0.004	0.009 ± 0.006	0.007 ± 0.005	0.008 ± 0.005
	Post-test	0.007 ± 0.006	0.009 ± 0.007	0.005 ± 0.005	0.007 ± 0.007	0.006 ± 0.004	0.006 ± 0.005
BF MEP_50_ (mV.s)	Baseline	0.007 ± 0.006	0.009 ± 0.008	0.007 ± 0.006	0.007 ± 0.003	0.007 ± 0.006	0.006 ± 0.004
	Post-test	0.006 ± 0.006	0.008 ± 0.007	0.004 ± 0.002	0.005 ± 0.003	0.005 ± 0.003	0.004 ± 0.002

Note: The shaded boxes indicate that there was a significant time effect between baseline and post-test measures; # means significantly different from baseline (interaction effect).
